# Fast automated energy changes at synchrotron radiation beamlines equipped with transfocator or focusing mirrors

**DOI:** 10.1107/S1600577522001084

**Published:** 2022-02-15

**Authors:** Sergey Stepanov, David Kissick, Oleg Makarov, Mark Hilgart, Michael Becker, Nagarajan Venugopalan, Shenglan Xu, Janet L. Smith, Robert F. Fischetti

**Affiliations:** a Advanced Photon Source, Argonne National Laboratory, 9700 South Cass Avenue, Bldg. 436D, Argonne, IL 60439, USA; b Life Sciences Institute, University of Michigan, 210 Washtenaw Avenue, Ann Arbor, MI 48109, USA

**Keywords:** synchrotron radiation, beamline automation, macromolecular crystallography

## Abstract

Algorithms and procedures to fully automate retuning of synchrotron radiation beamlines over wide energy ranges are discussed. The discussion is based on the implementation at the National Institute of General Medical Sciences and the National Cancer Institute Structural Biology Facility at the Advanced Photon Source. When a user selects a new beamline energy, software synchronously controls the beamline monochromator and undulator to maintain the X-ray beam flux after the monochromator, preserves beam attenuation by determining a new set of attenuator foils, changes, as needed, mirror reflecting stripes and the undulator harmonic, preserves beam focal distance of compound refractive lens focusing by changing the In/Out combination of lenses in the transfocator, and, finally, restores beam position at the sample by on-the-fly scanning of either the Kirkpatrick–Baez mirror angles or the transfocator up/down and inboard/outboard positions. The sample is protected from radiation damage by automatically moving it out of the beam during the energy change and optimization.

## Abstract 2

Procedures for fully automated X-ray energy changes at undulator-based synchrotron radiation beamlines with Kirkpatrick–Baez focusing mirrors or a long transfocator consisting of compound refractive lenses are presented. The procedures maintain beam intensity after the monochromator, beam position at the sample, beam attenuation, and the beam focusing distance.

## 1. Introduction

Reconfiguring synchrotron radiation beamlines from one X-ray energy to another is a frequent requirement in many types of synchrotron experiments. For example, in macromolecular X-ray crystallography (MX) it is often required to change the energy by several keV in order to switch from one absorption edge in the sample to another. Despite the apparent simplicity, changing beamline energy is a complex task involving multiple beamline components, and a failure at any step may lead to loss of beam intensity, position, or focus. Searching for lost beam may take hours. Therefore, it is critical for this process to be reliable and fully automated so that users can perform it without beamline staff involvement. One example is the case of MX beamlines where typical beam time allocated for an experiment ranges between 6 and 24 h and, in most cases, users and lately, due to the pandemic, even support staff, are remote. Obviously, for such short experiments, changing beamline energy should be as simple, smooth, and fast as possible. A number of MX and some non-MX beamlines have reported automating energy changes as a part of experiment automation (Abola *et al.*, 2000 ▸; Arzt *et al.*, 2005 ▸; Owen *et al.*, 2016 ▸; Aragão *et al.*, 2018 ▸; Mangold, 2018 ▸; Schneider *et al.*, 2021 ▸). However, very few implementation details of the energy changes were provided and in some cases only a partial automation or full automation in a limited energy range was mentioned (Arzt *et al.*, 2005 ▸; Aragão *et al.*, 2018 ▸). The aim of this paper is to fill this gap and primarily discuss the algorithms for efficient automated retuning of synchrotron radiation beamlines over a wide energy range by up to dozens of keV. Although the paper is based on the automations implemented at the National Institute of General Medical Sciences and the National Cancer Institute (GM/CA) MX beamlines at the Advanced Photon Source (APS) and uses that implementation in the examples, we attempt to abstract the discussed algorithms from particular implementation. Therefore we provide the minimum beamline specifications only as needed for understanding. Observing the other (non-MX) APS beamlines which have all switched to remote operations because of pandemics, automating energy changes currently presents value for the broader synchrotron radiation community than just MX and deserves consideration unlinked to the specifics of MX experiments.

**Figure 1:**
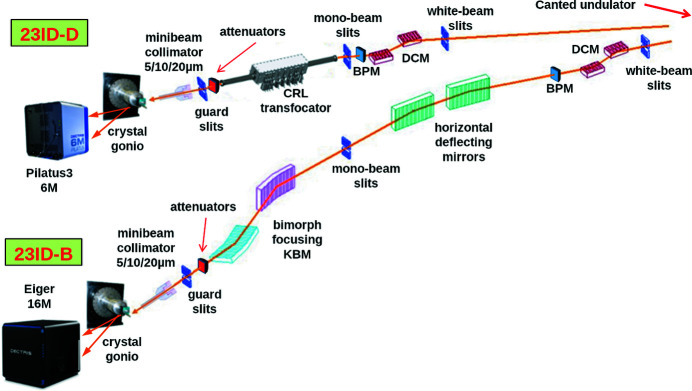
Layout of the GM/CA canted undulator beamlines at the APS. DCM, double-crystal monochromator; BPM, beam position monitor used in maximizing beam flux (intensity feedback); CRL, compound refractive lens; and KBM, Kirkpatrick–Baez mirrors.

GM/CA at APS operates two canted undulator MX beamlines (Fischetti *et al.*, 2007 ▸). The beamline layout is shown in Fig. 1 ▸. Both beamlines are equipped with constant exit height double-crystal monochromators (DCMs). To provide a wide energy range with constant beam height, the second crystal is shifted along the beam synchronously with Bragg angle changes. In addition, the second crystal is equipped with motorized motions for adjusting the pitch and roll angles. Small pitch angle adjustments in the range ±100 µrad can also be achieved with a fast piezo actuator. The latter is used in the feedback system maximizing photon beam intensity at a beam position monitor after the monochromator (Fischetti *et al.*, 2004 ▸, 2007 ▸). The concept of and name for beamline intensity feedback were introduced by Krolzig *et al.* (1984 ▸) and used at many beamlines, for example at Diamond (Bloomer *et al.*, 2013 ▸). The design of the GM/CA monochromators is typical for many facilities and is currently the most commonly used at MX beamlines (Owen *et al.*, 2016 ▸). The GM/CA beamlines are designed for energy ranges 3.5–20 keV (23ID-B) and 6–35 keV (23ID-D). The smaller energy range for 23ID-B is due to additional horizontal deflecting mirrors that separate the beams from the canted undulator sources.

Beamline 23ID-B has a set of bimorph Kirkpatrick–Baez focusing mirrors (KBMs) and two horizontal deflecting mirrors. The mirrors have three parallel stripes for use depending on the X-ray energy: plain Si surface, Rh-coated or Pt-coated.

**Figure 2:**
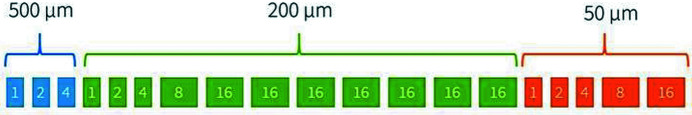
CRL transfocator layout at beamline 23ID-D. The numbers in the boxes show the number of lenses in each lens block and the numbers above indicate their radius of curvature. X-rays propagate from left to right.

Beamline 23ID-D was recently upgraded: a compound refractive lens (CRL) transfocator with paraboloid lenses instead of a KBM system is used to provide a 2D micro-focusing capability. CRLs were introduced to synchrotron radiation beamlines by Snigirev *et al.* (1996 ▸). To preserve the CRL focal point when changing beamline energy they needed to be shifted upstream or downstream. A transfocator is an array of CRLs that can be placed in and out of the beam to change the overall focal distance. It simplifies maintaining focus at the same point when changing energy by reducing the required device shift (Vaughan *et al.*, 2011 ▸). The GM/CA transfocator is located at 70.5 m from the undulator source and focuses at the sample position, which is 1538.8 mm downstream from the trans­focator center. The device contains 19 groups of paraboloid Be lenses (165 lenses total) of which 14 groups (a total of 134 lenses of 200 µm and 500 µm diameter) are in use below 20 keV; the other 5 groups of 50 µm are added at higher energies (Fig. 2 ▸). With 19 groups, the length of the CRL stack is 498 mm while with 14 groups it is 375 mm. While CRL transfocators may provide a cleaner focus than KBMs, they introduce an additional complexity to beamline energy changes because, unlike focusing mirrors, CRLs are dispersive devices and their focusing distance changes with X-ray energy. To compensate for that, one needs to find the best matching combination of the CRL blocks In/Out of the beam and to shift the transfocator along the beam to accommodate the remaining difference. While transfocators are currently used at many beamlines, not only at ESRF where they were introduced but also for example at Petra III (Zozulya *et al.*, 2012 ▸) and NSLS II (Schneider *et al.*, 2021 ▸), the GM/CA transfocator presents an additional complexity for calculating its focal distance because it is one of the longest CRL transfocators to date with the device length comparable with the focusing distance (498 mm versus 1538.8 mm).

**Figure 3:**
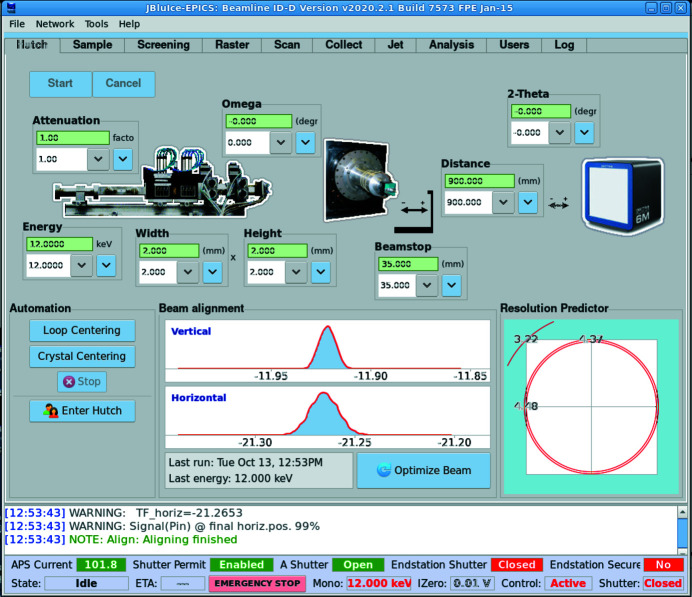
*JBluIce* Hutch tab with controls for energy changes. This graphical user interface is provided to the GM/CA users who can dial the required energy in the input field at the left and then click the Start button. All further steps are performed automatically.

Identical software carries out beamline energy changes at both 23ID beamlines. It is configurable through a mySQL database and command line parameters supplied by the graphical user interface. Beamline users access energy changes as a simple energy input (Fig. 3 ▸) on the Hutch tab of the *JBluIce* data acquisition program (Stepanov *et al.*, 2011 ▸). Both *JBluIce* and all software for energy changes are open source and available for download from the JBluIce-EPICS source depository (JBluIce-EPICS, 2021 ▸).

Below we describe step-by-step the procedures for changing energy implemented in the software. In some cases, several alternative algorithms are available to accommodate differing beamline characteristics.

## 2. Synchronization of monochromator and undulator

The first step of reconfiguring the beamline to a new energy is to retune the Bragg angle of the monochromator crystal and the gap of the undulator insertion device (ID) so they both permit this energy. A key point here is to keep these two devices roughly synchronized during the energy change so that the beamline intensity feedback stays locked. The beamline intensity feedback is a software or hardware tool aimed at maintaining the second monochromator crystal parallel to the first crystal so that the monochromator passes a beam of maximum intensity. It continuously dithers (slightly tweaks) the second crystal angle with a piezo actuator, checks for an increase or decrease in the beam flux after the monochromator, and then drives the angle in the direction of greater intensity. GM/CA has implemented both software and hardware intensity feedback (Fischetti *et al.*, 2007 ▸). The latter is based on the lock-in amplifier SR810 by Stanford Research Systems. It is significantly faster, but may require different tuning for different energy ranges as the Bragg peak narrows with increasing X-ray energy. Thus, when going to high energies (above ∼20 keV at GM/CA) one may want to switch to using a slower, but more robust, software feedback.

When the ID and the monochromator are tuned to different X-ray energies, the beam flux after the monochromator becomes low, and the feedback naturally fails, which then requires searching for the beam with the second crystal. This situation can arise because the ID retunes to a different energy faster than the monochromator and also because the monochromator retunes with constant speed over the Bragg angle change and the ID over the gap change, but neither over the energy change. Thus, three modes of driving the monochromator and ID have been implemented and can be selected in the beamline software configuration depending on the size of the energy change and the feedback system in use.

### 2.1. ID scan mode

This is the fastest mode. Based on the time required to move the monochromator from the old to the new Bragg angle, we reduce the speed of changing the ID gap to match and then start both devices synchronously. This method is optimal when the energy changes are relatively small, as then the non-linearity of monochromator and ID speeds over energy is not critical. At the GM/CA beamlines typically these are energy changes up to ∼1 keV in the energy range 10–16 keV. At other facilities these limits may be different depending on the ID parameters and selected Bragg reflection.

### 2.2. ID steps mode

This mode is almost as fast as the ID scan mode, adding only a few seconds to the energy change. The monochromator runs from the old to the new energy continuously. The ID speed is not reduced. Software monitors the monochromator energy while it is changing and requests the ID to move to a setting for an X-ray energy 100 eV ahead of the current value. As soon as the ID arrives, it is requested to go 100 eV ahead of the current monochromator energy, and so on, until both devices arrive at the target energy. Using small 100 eV steps keeps the ID always tuned within the width of an undulator harmonic. This method works well for fast hardware feedback that can cope with the monochromator dynamic intensity variations caused by some asynchronization between the monochromator and the ID.

### 2.3. Multi-step mode

This mode is slower, but is more reliable for slower intensity feedback systems such as the software feedback at GM/CA. The energy change interval is split into relatively small (∼250 eV) segments and then both monochromator and ID run in these steps; small pauses between steps allow the intensity feedback to lock. As each 250 eV step adds several seconds overhead, an overhead per 1 keV energy change is about 6–8 s. At the GM/CA beamline the choice of 250 eV works well over the whole energy ranges 6–35 keV and 3.5–20 keV at 23ID-D and 23ID-B, respectively, but one may need to adjust the step size if applying the multi-step energy changes algorithm at another facility.

### 2.4. ID harmonic and monochromator Bragg reflection changes

Some energy changes require changing the undulator harmonic as the minimum or maximum ID gap is reached. At the GM/CA beamlines this occurs at 15 keV, 24 keV and 34 keV, which are the limits for the first, third and fifth harmonics, respectively. When the system reaches the threshold for a harmonic change, the energy change and the intensity feedback are paused and the ID is commanded to switch to the next harmonic. Once the switch is complete, the energy change is resumed.

At high energies (above 20 keV in the case of GM/CA beamlines) the monochromators can no longer use the 111 Bragg reflection because the second crystal translation reaches the travel limit and the monochromator needs to be switched to the 333 Bragg reflection. The procedure is similar to the ID harmonic switches: the energy change and the intensity feedback are paused and the monochromator is commanded to switch to 333.

### 2.5. Preservation of attenuation

At GM/CA, the beam attenuation box contains 16 Ag and Al foils that can be inserted into or removed from the beam path to provide the requested attenuation. The box is located downstream of the focusing optics. When the X-ray energy changes, the efficiency of the foils also changes, and a new foil combination needs to be found in order to maintain the beam attenuation. This is recalculated in the loop carrying out the energy change in all three modes. Without this recalculation, the attenuation could be greatly reduced when driving to a higher energy, and the sample would receive an unintended high dose if the energy is changed when the sample shutter is open.

### 2.6. Switching mirror reflecting stripes

When moving to a higher energy, the angle of X-ray total external reflection from the beamline focusing and deflecting mirrors (Fig. 1 ▸) may become smaller than the angle of the mirrors to the beam, and then the mirrors may stop passing the beam. Since changing the mirror angle is problematic as it would require rotating all beamline components downstream of the mirror, the mirror has a cutoff energy above which it cannot be used. A common solution to this problem is to install mirrors with stripes of different coating materials corresponding to different cutoff energies (Owen *et al.*, 2016 ▸; Aragão *et al.*, 2018 ▸; Schneider *et al.*, 2021 ▸). Coatings with higher *Z* numbers provide higher cut-off photon energies, which allow the mirrors to continue reflecting. At GM/CA the mirrors have three stripes: bare Si, Rh-coated and Pt-coated. When moving to a higher energy, the mirrors can be switched from Si to the higher-*Z* Rh, and then to the even higher-*Z* Pt. At the GM/CA beamline 23ID-D this switching is required at 9.35 and 18.5 keV, respectively. At beamline 23ID-B, which additionally has two horizontal deflecting mirrors, the switching is required at 9.35 and 15 keV. These values are stored in the beamline configuration database. When moving to lower energy, the mirrors are switched back from Pt to Rh and Si to avoid passing higher X-ray harmonics from the undulator. In principle, it is better to change the mirror stripes to a coating with higher-*Z* element before an energy increase and back to a lighter or no coating after an energy decrease. This assures that the mirrors pass an X-ray beam throughout the energy change process. Since the mirrors are downstream of the feedback detection system, it is not required to synchronize the stripes changes with monochromator motion.

## 3. Transfocator server

When a beamline includes a CRL transfocator, which is a dispersive focusing device, it needs to be reconfigured to place a different CRL combination in the beam in order to preserve the focus. At GM/CA beamline 23ID-D, this is implemented with the help of a software server known as the Transfocator Server (TS). The focal distance of a lens is given by the lensemaker’s equation, which in the X-ray range and the case of a symmetric lens reads

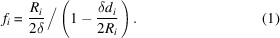

Here *R*_*i*_ is the lens radius (500, 200, or 50 µm in the case of GM/CA lenses), *d*_*i*_ is the maximum lens thickness and δ is the decrement of the refractive index *n*: *n* = 1 − δ. In the X-ray range the values of δ are very small, δ ≃ 10^−5^, and then the second term in the denominator of equation (1) can be neglected for any realistic lens thicknesses, which are typically ∼1 mm or even for the CRL groups with the total ‘thickness’ up to ∼30 mm. It corresponds to a ‘thin’ lens approximation and thus we treat each of 19 lens groups (Fig. 2 ▸) as a ‘thin’ lens with a focal length of

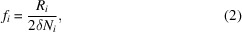

where *N*_*i*_ is the number of lenses (1, 2, 4, 8, or 16). The values of δ are tabulated as a function of X-ray energy, and, for a given beamline energy, the TS interpolates δ from the tabular data. The overall focal distance of the transfocator is calculated by the recursive equation (Bruls, 2015 ▸),

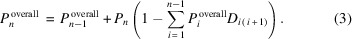

Here *P*_*i*_ = 1/*f*_*i*_ is the optical power of each lens block, *D*_*i*(*i*+1)_ is the distance between the lens blocks, and the summation is over all lens blocks in the beam. The recursive calculation starts with two lenses and works over the stack until all lenses are accounted for. The overall focal distance of the transfocator 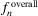 = 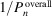 is counted from the rear (downstream) principal optical plane *P*_R_ of the current lens stack (Bruls, 2015 ▸),

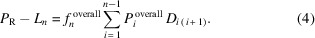

Here *L*_*n*_ is the position of the last (most downstream) lens block inserted into the beam.

The TS monitors the X-ray energy permitted by the monochromator and the In/Out states of all lens blocks. It calculates the overall focal distance and sums it with the offset of the transfocator box from its nominal distance from the X-ray source. Once the beam is focused at a specific position for a given energy, this focal distance should remain unchanged when changing the beam energy, and is saved as a setpoint. After the beam energy is changed, the TS is commanded to match the setpoint. First, it applies equations (3)–(4) to all possible combinations of CRL blocks (2^19^ or 2^14^ combinations at GM/CA) and sorts them over the difference with the setpoint value. Then, the closest combination is selected and the remaining difference with the setpoint is compensated by shifting the transfocator box up- or down-stream. Although the number of combinations is vast, the calculation is simple and modern workstations perform it with insignificant delay (under 1–2 s). To improve the speed, optical powers *P*_*i*_ can be cached.

We have also implemented a second TS mode aimed at reducing the beam attenuation due to absorption by the CRLs. In this mode the TS is supplied with a tolerance interval from the focal setpoint, for example ±25 mm, within which the number of lenses in the beam can be reduced. Then, instead of using the best focal distance match, the TS selects the first *N* elements of the sorted array where the mismatch is within the tolerance interval. The selected short array is then sorted over the number of lenses and the candidate with the minimum number is selected. Any mismatch is compensated by shifting the transfocator box.

**Figure 4:**
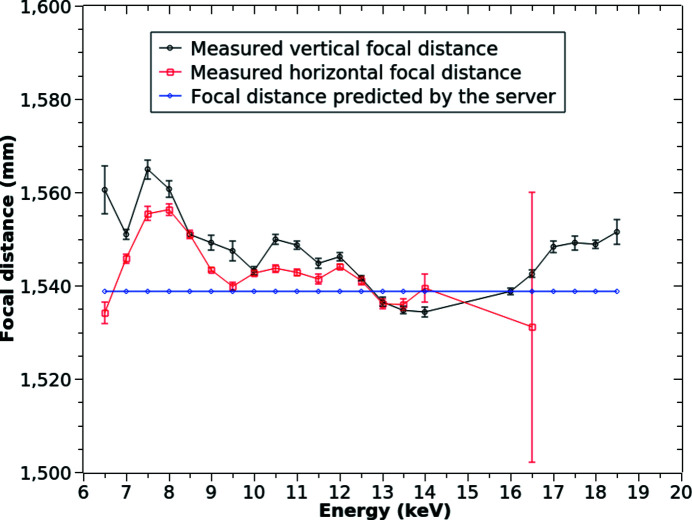
Predicted *versus* measured focal distances in the energy range from 6.5 to 18.5 keV. The horizontal measurements above 16.5 keV were too noisy and the error bars exceeded the mean deviations; therefore they are excluded. The magnitude of differences (<25 mm) is expanded by the choice of *Y*-scale. Compared with the 1538.8 mm focal distance, which is the transfocator setpoint, they are minor (less than 1.69%).

Fig. 4 ▸ presents the results of testing the TS with knife-edge scans. First, the beam was focused at the knife-edge when the beamline energy was 12.09 keV. The focal distance calculated by the TS (1538.8 mm) was saved as a setpoint. Then the beamline was reconfigured to different energies with the TS preserving the best focus match to the setpoint. At each new energy we performed multiple knife-edge scans. One could either shift the transfocator downstream or slightly vary the energy to find the best focus measured by the knife-edge scan. For this test, we chose the simpler path of varying the energy by changing the monochromator crystal angle and the ID gap. The energy deviations corresponding to the best focus were then recalculated into the focal distance errors. As demonstrated in Fig. 4 ▸, the focal distance errors are small over a wide energy range from 6.5 keV to 18.5 keV: for the vertical focus the error does not exceed 1.69% and for the horizontal 1.14%. Small correlated deviations of both vertical and horizontal focal distances from the prediction may be due to minor variations of the lens thickness or deviations from the parabolic shape which are beyond the metrology access. They may also be due to the limitation of the thin lens approximation [see equations (1)–(2)]: when δ ≃ 10^−5^, *d* = 30 mm, and *R* = 200 µm, then δ*d*/2*R* = 0.75%, which is comparable with the observed differences. Further minor differences between measured horizontal and vertical focal distances may be due to the asymmetric shape of the APS beam (the beam is ten times wider horizontally) and to the size difference between horizontal and vertical white-beam slits. When the slits are partially closed, the source becomes a mix of the ID emission point and the slit which are at 70.5 m and 43.3 m away from the CRL transfocator, respectively.

## 4. Aligning beam position to the sample

Changing the X-ray energy may cause vertical and horizontal shifts of the beam at the sample. Although, as with most ID beamlines, GM/CA uses constant-exit monochromators with cryo-cooled first and second crystals, various factors may contribute to the shifts including slight changes in the beam direction after the ID, a sub-optimally tuned roll angle of the monochromator second crystal, variations in the grazing angles of the beam to the mirrors after the reflecting stripe change, *etc*. Here, the beam deviations are a few micrometres at a beamline length greater than 70 m. To compensate for these possible lateral shifts, the automated energy change software may perform vertical and horizontal scans to re-center the beam. The decision whether to perform an automatic scan is taken when the Bragg angle is altered by more than a configurable threshold (typically ∼1°), when the undulator harmonic is changed, or when the mirror reflecting stripes are changed. In addition, users can request an optimization at any time. The optimization software scans the beam through a small 10 µm or 5 µm collimator (Fischetti *et al.*, 2009 ▸) that is 30 mm upstream of the sample position and then brings it to the center. The results are displayed on the Hutch tab of *JBluIce* (Fig. 3 ▸).

Different scans are performed for a beamline equipped with KBMs or with a CRL transfocator. In the case of KBMs, we perform on-the-fly scans of the piezo voltage that changes the grazing angle of the vertical or horizontal KBM relative to the beam. The beam intensity after the collimator is recorded while the voltage on the piezo is gradually increased. Then, the piezo is driven to the peak position. In the case of the transfocator we found it most efficient to perform on-the-fly scans of servo motors that change the transfocator vertical and horizontal positions rather than the respective tilt angles of the transfocator box. This procedure may change if a mirror is added to the beamline optical path.

Since both GM/CA beamlines are equipped with on-axis microscopes, in principle one could replace the scanning by bringing a YAG crystal into the beam and using the beam-spot image on the YAG captured by on-axis microscope to correct the respective grazing angles of the mirrors or lateral positions of the transfocator. However, we found this method to be less accurate than scanning. Additionally, the need to dismount the sample and mount a YAG crystal voids potential time savings.

At GM/CA, the goniometer is equipped with a pin-base sensor. When a pin is detected, which is normally a sample, the software may use one of the configurable options (auto-shift or user prompt) to preserve the sample. The sample is returned to the in-beam position on the goniometer after beam alignment.

## 5. All steps in action

**Figure 5:**
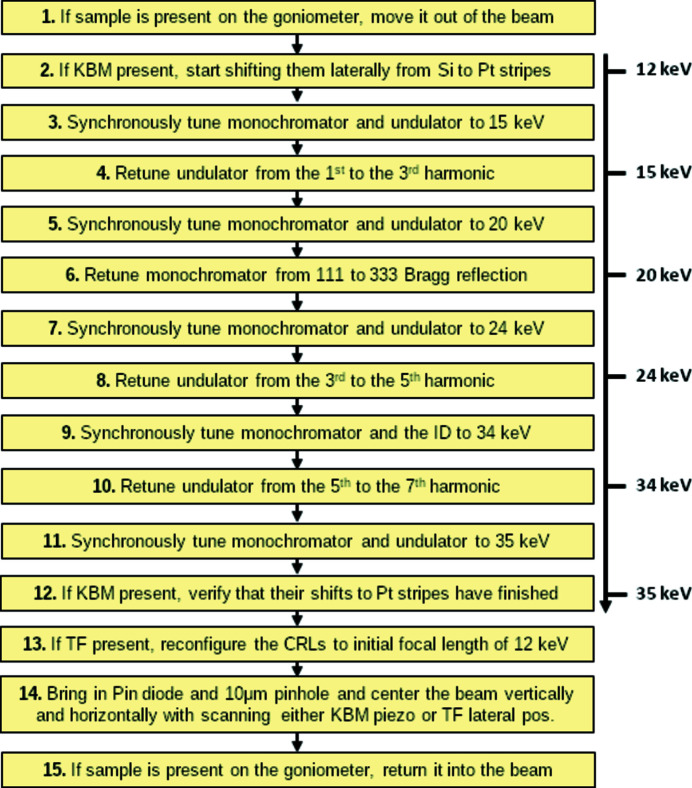
Diagram for retuning the beamline from 12 to 35 keV. At steps 3, 5, 7, 9, and 11, the intensity feedback maintains beam flux and the beam attenuation is preserved by changing the In/Out state of foils; at steps 4, 6, 8, and 10, the intensity feedback is paused.

Fig. 5 ▸ presents a diagram for retuning beamline 23ID-D from 12 keV to 35 keV. During this transition the undulator harmonic is changed from first to third, then to fifth and seventh, and the Bragg reflection is switched from 111 to 333. Software starts by removing the sample from the beam and initiating the lateral mirrors motion from the Si to the Pt reflecting stripes (when the mirrors are present). Then it implements five intervals of synchronously retuning the monochromator and the undulator. In this example the multistep algorithm (Section 2.3) was used. At these intervals the intensity feedback is turned on to maintain maximum beam flux after the monochromator. The attenuation is also maintained (Section 2.5). Between intervals, the feedback is turned off and either the ID harmonic or the Bragg reflection is changed. Once the final energy of 35 keV is reached, software verifies that the mirrors (if present) have finished changing the reflecting stripes and reconfigures the transfocator (if present) with the help of the transfocator server. If maintaining the focal distance during energy changes is needed, we can implement continuous refocusing with the transfocator server in the same way as the attenuation is maintained. Finally, software aligns the beam and returns the sample to the beam center.

The entire process of reconfiguring the beamline from 12 to 35 keV including 2D alignment takes under 12 minutes. A large portion of this time is consumed by changing the ID harmonics and especially the Bragg reflection. Smaller energy changes consume shorter time. For example, retuning from 7 to 12 keV takes 4 min, from 12 to 16 keV which involves one ID harmonic change at 15 keV takes 6 min, and from 12 to 13 keV consumes only 25 s.

## 6. Conclusions

We present procedures for automated reconfiguring of synchrotron beamlines to new energies. They cover various aspects of the process including preserving beam flux after the monochromator, beam attenuation, centered position at the sample, and focal distance. In this presentation we concentrated on ideas and algorithms rather than technical details in order to be useful to the broader synchrotron radiation community. All software is open source and available for download from the JBluIce-EPICS source depository (JBluice-EPICS, 2021 ▸). The scripts that perform energy changes and beam alignment as well as the transfocator server are standalone and can be used separately from the *JBluIce* beamline control and data acquisition graphical user interface. Therefore they can be relatively easily adapted at other facilities once their hardware-specific communications are changed.

## References

[bb1] Abola, E., Kuhn, P., Earnest, T. & Stevens, R. C. (2000). *Nat. Struct. Biol.* **7**, 973–977. 10.1038/80754 11104004

[bb2] Aragão, D., Aishima, J., Cherukuvada, H., Clarken, R., Clift, M., Cowieson, N. P., Ericsson, D. J., Gee, C. L., Macedo, S., Mudie, N., Panjikar, S., Price, J. R., Riboldi-Tunnicliffe, A., Rostan, R., Williamson, R. & Caradoc-Davies, T. T. (2018). *J. Synchrotron Rad.* **25**, 885–891. 10.1107/S1600577518003120 PMC5929359 29714201

[bb3] Arzt, S., Beteva, A., Cipriani, F., Delageniere, S., Felisaz, F., Förstner, G., Gordon, E., Launer, L., Lavault, B., Leonard, G., Mairs, T., McCarthy, A., McCarthy, J., McSweeney, S., Meyer, J., Mitchell, E., Monaco, S., Nurizzo, D., Ravelli, R., Rey, V., Shepard, W., Spruce, D., Svensson, O. & Theveneau, P. (2005). *Prog. Biophys. Mol. Biol.* **89**, 124–152. 10.1016/j.pbiomolbio.2004.09.003 15910915

[bb4] Bloomer, C., Dent, A., Diaz-Moreno, S., Dolbnya, I., Pedersen, U., Rehm, G., Tang, C. & Thomas, C. (2013). *J. Phys. Conf. Ser.* **425**, 042010.

[bb5] Bruls, G. J. C. L. (2015). *Optik*, **126**, 659–662.

[bb6] Fischetti, R., Stepanov, S., Rosenbaum, G., Barrea, R., Black, E., Gore, D., Heurich, R., Kondrashkina, E., Kropf, A. J., Wang, S., Zhang, K., Irving, T. C. & Bunker, G. B. (2004). *J. Synchrotron Rad.* **11**, 399–405. 10.1107/S0909049504016760 15310956

[bb7] Fischetti, R. F., Xu, S., Yoder, D. W., Becker, M., Nagarajan, V., Sanishvili, R., Hilgart, M. C., Stepanov, S., Makarov, O. & Smith, J. L. (2009). *J. Synchrotron Rad.* **16**, 217–225. 10.1107/S0909049508040612 PMC2725011 19240333

[bb8] Fischetti, R. F., Yoder, D. W., Xu, S., Stepanov, S., Makarov, O., Benn, R., Corcoran, S., Diete, W., Schwoerer-Boehing, M., Signorato, R., Schroeder, L., Berman, L., Viccaro, P. J. & Smith, J. L. (2007). *Ninth International Conference on Synchrotron Radiation Instrumentation*, edited by J.-Y. Choi and S. Rah, pp. 754–757. New York: American Institute of Physics.

[bb9] JBluice-EPICS (2021). * *JBluIce*-EPICS source depository*, https://www.gmca.aps.anl.gov/jbluice-epics/.

[bb10] Krolzig, A., Materlik, G., Swars, M. & Zegenhagen, J. (1984). *Nucl. Instrum. Methods Phys. Res.* **219**, 430–434.

[bb11] Mangold, S. (2018). *J. Synchrotron Rad.* **25**, 960–966. 10.1107/S1600577518007518 PMC6038605 29979156

[bb12] Owen, R. L., Juanhuix, J. & Fuchs, M. (2016). *Arch. Biochem. Biophys.* **602**, 21–31. 10.1016/j.abb.2016.03.021 PMC5505570 27046341

[bb13] Schneider, D. K., Shi, W., Andi, B., Jakoncic, J., Gao, Y., Bhogadi, D. K., Myers, S. F., Martins, B., Skinner, J. M., Aishima, J., Qian, K., Bernstein, H. J., Lazo, E. O., Langdon, T., Lara, J., Shea-McCarthy, G., Idir, M., Huang, L., Chubar, O., Sweet, R. M., Berman, L. E., McSweeney, S. & Fuchs, M. R. (2021). *J. Synchrotron Rad.* **28**, 650–665. 10.1107/S1600577520016173 PMC7941291 33650577

[bb14] Snigirev, A., Kohn, A., Snigireva, V. & Lengeler, I. (1996). *Nature*, **384**, 49–51.

[bb15] Stepanov, S., Makarov, O., Hilgart, M., Pothineni, S. B., Urakhchin, A., Devarapalli, S., Yoder, D., Becker, M., Ogata, C., Sanishvili, R., Venugopalan, N., Smith, J. L. & Fischetti, R. F. (2011). *Acta Cryst.* D**67**, 176–188. 10.1107/S0907444910053916 PMC3046456 21358048

[bb16] Vaughan, G. B. M., Wright, J. P., Bytchkov, A., Rossat, M., Gleyzolle, H., Snigireva, I. & Snigirev, A. (2011). *J. Synchrotron Rad.* **18**, 125–133. 10.1107/S0909049510044365 PMC3267637 21335897

[bb17] Zozulya, A. V., Bondarenko, S., Schavkan, A., Westermeier, F., Grübel, G. & Sprung, M. (2012). *Opt. Express*, **20**, 18967–18976. 10.1364/OE.20.018967 23038536

